# Determinants of maternal and household social inequalities of dental caries among Senegalese children in the Department of Pikine

**DOI:** 10.11604/pamj.2023.44.23.31643

**Published:** 2023-01-12

**Authors:** Serigne Ndame Dieng, Aïda Kanouté, Sylvie Azogui-Levy, Pierre Lombrail

**Affiliations:** 1 Public Health Service, Institute of Odontology and Stomatology, Cheikh Anta Diop University, Dakar, Senegal,; 2 Training and Research Unit (UFR) of Odontology, University of Paris 7, Paris, France,; 3 Laboratory of Education and Promotion of Health (LEPS), University of Paris 13, Sorbonne Paris City, Bobigny, France

**Keywords:** Dental caries, children, social determinant, mother, household

## Abstract

**Introduction:**

the problematic of social Inequalities in oral health remains a global concern; it constitutes evidence of social injustice. The present work aims to study the determinants of maternal and household social inequalities of children´s dental caries in Pikine.

**Methods:**

cross-sectional epidemiological survey has been conducted in the department of Pikine, Senegal on 315 children aged 3 to 9 and their mothers. The clinical data on children´s caries have been obtained by clinical examination and the socio-economic data by a questionnaire submitted to mothers. Pearson chi-square and trend tests as well as a logistic model were used in the data analysis.

**Results:**

the prevalence of dental caries in children was 64.8% and the mixed decayed, filled, missing (DFM) index was 2.5 (±2.7). The trend test showed significant inequalities in the prevalence of dental caries according to level of studies (p<0.001), profession (p<0.010) and contacts frequency (p<0.001) of mothers; the level of wealth (p<0.001) and structure (p<0.005) of households. According to the logistic regression model, the level of secondary or university education [OR (IC 95%) = 0.59 (0.33'>OR (IC 95%) = 0.59 (0.33-0.93)] or social network dynamism [OR (IC 95%) = 0.32(0.15'>OR (IC 95%) = 0.32(0.15-0.67)] of mothers; as well as wealthy families [OR (IC 95%) = 0.23(0.08'>OR (IC 95%) = 0.23(0.08-0.64) were associated to fewer risks of dental caries among children.

**Conclusion:**

some socio-economic characteristics of the mother and the household social conditions are identified as determinants of dental caries social inequalities in Children. Proportionate universalism may be a good approach to reduce this problematic in Pikine.

## Introduction

The problematic of social inequalities in health (SIH) remains a global concern because it constitutes evidence of social injustice [1]. The SIH, are in terms of health, the consequences of the way in which resources and constraints are unfairly allocated to the population [2,3]. The higher somebody´s social position is, the less they have constraints and the more resources they get, the better their health is [4,5]. In the field of oral health, this ascertainment can be testified in the fact that there is a social gradient of dental caries in the population [6,7], especially among kids. Indeed, Folayan *et al*. reported in 2021 that Nigerians adolescents of higher socio-economic status had significantly lower prevalence of caries than those of lower status (OR=0.40; IC à 95% 0.17, 0.91) [6]. In these last years, several theoretical models had been conceptualized in order to explain the mechanisms through which the social determinants have affected oral health, particularly that of children [7-9]. Fisher-Owens *et al*. proposed a model showing a multidimensional approach in which the influences are not isolated, but they are rather in intricate and hierarchical interactions. They stated that the influential factors of children´s oral health are individual, family and community order [10]. Children live in families and the families are integrated into the communities. Therefore, the risk of oral disease of a child can neither be isolated from the risk of the family disease nor from that of the community [11].

The family environment is significantly associated with onset of social inequalities of children´s dental caries. Family social conditions put the child in some positions of unequal vulnerability as far as oral diseases are concerned [12]. That is the same case for the individual characteristics of family members and particularly of the mother, which are linked to children´s oral health status [7,13]. In many Sub-Saharan African countries, the households face the social precarity with its implications in oral health [14]. This situation makes it difficult to access healthcare and prevention services, especially fluoride products. The World Health Organization (WHO) associated this high prevalence of children´s caries in Africa to the weaknesses of care offer and inaccessibility of fluoridated products [15]. The social precarity exposes also the populations, especially the children to an unchecked, unbalanced and a sugary food [16]. Joury *et al*. reported a frequency of consumption of sweet food higher in poor groups among Syrian´s children [17]. Besides, the level of knowledge of mothers can be an explanatory factor to inequalities in children tooth decays [18]. The literature suggests a linear link between mothers´ oral health knowledge and attitude and the risk of children´s dental caries [19]. In Senegal, from small portions of data, social inequalities of caries have been observed among children. A prevalence of 78% of tooth decay and 55% have been found in preschool children aged 3 to 6 and living respectively in the suburbs and in residential districts of Dakar [20]. Responses that essentially oriented to therapeutic care have so far failed to reduce SIH. The social determinant's of health approach can be explored as a complement to the curative response. It consists of taking action upstream through public policies on modifiable social health factors. The emphasis should be laid on the central role of the mother and family social conditions. To do this, documenting the family and maternal determinants of children´s oral health becomes relevant; these determinants would be associated with the risk of the occurrence of social inequalities in children's caries. The objective of this work is to determine maternal and family determinants of social inequalities of children´s dental caries in Pikine.

## Methods

**Place, type and study population:** a cross-sectional epidemiological survey has been conducted from February to May 2015 in Pikine, one of the departments of Dakar. Pikine has 3 districts and 16 municipalities and is the most populated department of Senegal [21]. It is located in periphery (suburb) of Dakar and is confronted to social precarity [22]. The study population was constituted of children aged between 3 and 9 years old and their mothers. Children were included when they were residents of one of the selected municipalities for at least a year and when they had lived with their mothers since their birth. As for the mothers, they should accept their participation in the study and that of the child by signing a free and clear consent. The non-inclusion of children and their mothers was linked to the fact that they were carrying a disability or any other illness that could make data collection impossible.

**Sampling and study variables:** a multiple-stage sampling clustered was implemented. First, 9 municipalities of 16 had been drawn with at least half of the municipalities per districts. Secondly, houses to be investigated had been identified within the selected municipalities. From a sociological center, the turned bottle method had allowed to specify the direction to be chosen to start the investigation. The first chosen right-hand house constitutes the first one to be inquired. A three steps leap of houses had permitted to identify the following ones. Two houses in addition had been chosen at random in each house and two children of different mothers in addition in each house. The so-called “exploratory” method defined by the WHO for field investigation [23] had allowed defining the size sample. It suggests selecting 20 to 50 subjects by site according to the gravity of the illness. Based on a 68% prevalence of children´s caries in Pikine (20), the subjects´ number by site was estimated to 35. The sample size was thus: N= 35 x 9 =315 children, exactly of 315 peers´ children-mothers. The “dental caries” variable reflects any clinically cavity observable lesion. It has been evaluated from two indicators: prevalence (P) and the DMF /df (number of decayed, missing and filled teeth). This average index, written into lowercase (df where d=decayed, f= filled), is destined for temporary teeth, into uppercase (DMF where D= decayed, M=missing and O= filled) for permanent teeth [23]. Social characteristics of the mother have been approached by the following variables. The level of education which corresponds to the attended cycle, is categorized into illiterate or primary/secondary or university. Paid occupation is binary: yes/no; the profession is defined according to three categories: worker/intermediary profession/manager. The social networking corresponds to the mother´s social interactions with those around them. It is assessed in number of contacts (density) and the contacts' frequency (dynamism) [24,25]. The contacts number called then “social support” is divided into modalities from a median of 5 contacts into: weak support/strong support. The frequency is divided into three categories: less than once a month/ at least once a month/at least once a week. The age of the mother is given into years then categorized into two modalities according to the median age: at least 35 years old/ 35 years old at most. The level of wealth, the structure and density of the housing are the variables proxies of the housing social conditions. The level of wealth has been rated by establishing a list of the family assets. This list has been compared to a list of defined basic assets by referring to the indicators used in the poverty monitoring reports in Senegal. It distinguishes the level of wealth of the households: poor, average, wealth. The household structure is defined by the link of parenthood between household members and formed around the couple or a parent and their children [26]. It can be a nuclear, extended or reconstituted family. A nuclear family is composed of the couple with or without their children, an extended family is the nuclear family and other people with or without family relationships, and the reconstituted family is constituted of a married couple, children who are not from the couple and possibly other members. The density is defined by the relation between the number of family members and the available accommodating rooms for the family. It is binary: less dense/dense.

**Data collection:** the data have been collected by two examiners and one principal investigator (all dentists) who are calibrated to WHO standards [23]. The examiners´ inter-concordance estimated by the Kappa test on a sample of 20 persons from another city of the suburb of Dakar was 87.2%. The data on children´s dental caries were collected by a clinical examination at home with their mother´s presence. The mothers had given information through a questionnaire on their socio-economic characteristics and those of the households. The questionnaires have been administered into Wolof or French. The research was authorized by the National Ethical Committee for Health Research (NECHR) of the Senegalese ministry of health and social action under the reference 00050MSAS/DPRS/CNERS.

**Analysis plan:** the data was entered in Excel and analyzed with STATA 13. The descriptive analysis has been realized with percentages for the qualitative variables and with averages for the quantitative variables with their scattering parameters. A bivariate analysis with Pearson´s Chi-square test permitted to appreciate the strength of the relationship between each variable and tooth decay. The Chi-square trend test was used to verify the distribution of the prevalence between modalities of the variables. A logistic regression model was built to analyze the association between dental caries and the variables after adjustment, this association was expressed in OR with their confidence range of 95%. Age being a potential confounding factor, it has been integrated into the model in order to control it. The categories with the lowest sociologically rank were chosen as reference and the p-value fixed at 0.05.

## Results

**Figure 1:**
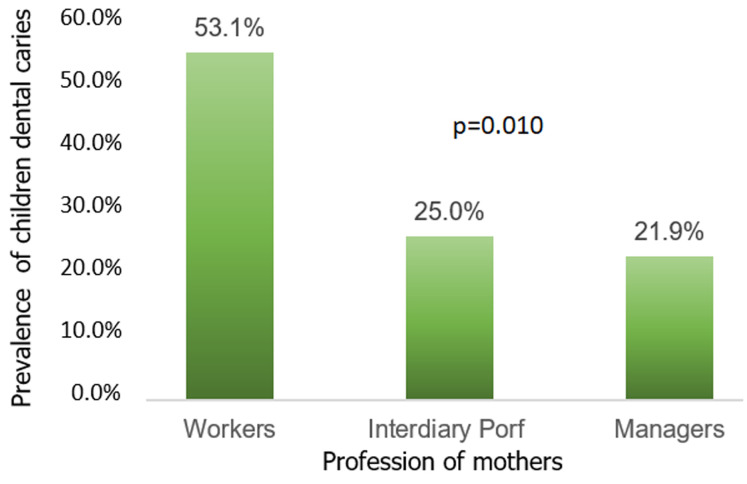
trends of dental caries prevalence in children by maternal characteristics

**Figure 2:**
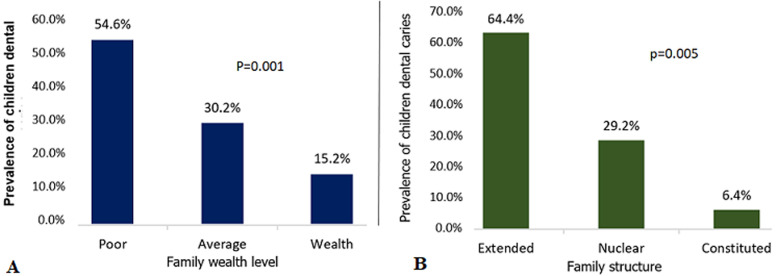
A, B) trends of dental caries prevalence in children by household characteristics

The sample consisted of 315 children aged 3 to 9 and their mothers aged 20 to 51. The children´s average age was 5±7 years old and that of their mothers 34±7years of age. They were in the majority literate (63.8%) but 31.7% only reached a secondary or university level. Fifty-two percent of the mothers (52%) had a paid occupation. Among these, 53.1% were workers and 21.9% managers. Regarding the social networking, more than once a week, 41.9% of mothers were in contact with those around them. Households were poor (54.6%) and extended type (64.4%). The median occupancy density was of 3 persons per room (with extremes of 1 to 7) and 65.7% of households had dwellings considered “dense” (Table 1). The prevalence of dental caries in Children was 64.8% and the combined DMF index of 2.5±2.7. In bivariate analysis, the prevalence was significantly unequal according to level of studies (p<0.001), profession (p<0.027) and contacts frequency (p<0.001) of mothers; the level of wealth (p<0.001) and structure (p<0.21) of the household as well as the density of housing (p<0.027). The trend test was significant for the same variables (Figure 1, Figure 2). The logistic regression model (Table 2) showed a significantly protective association against childhood caries if the mothers had a secondary or university studies level [OR (IC95%) =0.59(0.33-0.93)], a contact frequency of at least once a week [OR (IC 95%) =0.32(0.15-0.67) and they belonged to a wealthy family [OR (95%)=0.23(0.08-0.64)].

**Table 1:** distribution of mothers and households’ characteristics

Variables		
Mothers’ characteristics; N=315	N	%
**Age class**		
≤ 35 years old	188	59.7%
> 35 years old	127	40.3%
Age average into years (±SD)	40 (±8.8)	
**Studies level**		
None-primary	215	68.3%
Secondary or university	100	31.7%
**Paid occupation**		
No	151	47.9%
Yes	164	52.1%
**Profession**		
Working class	87	53.1%
Intermediary profession	41	25%
Executive	36	21.9%
**Social support**		
Weak supports	178	56.5%
Strong supports	137	43.5%
Averages of supports (±SD)	6 (±3)	
**Contacts’ frequency**	315	
Less than once/month	68	21.6%
At least once/month	116	36.8%
At least once/week	131	41.6%
**Household characteristics; N=315**		
**Structure of household**		
Extended	203	64.4 %
Nuclear	92	29.2 %
Reconstituted	20	6.4 %
**Level of wealth**		
Poor	172	54.6 %
Average	95	30.2 %
Wealth	48	15.2 %
**Density of occupation housing**		
Less dense	108	34.3%
Dense	207	65.7%

**Table 2:** distribution of children’s dental caries prevalence and logistical model

Variables	Numbers (prevalences)	decayed, filled, missing/decayed, filled (SD)	ORna (IC 95%)	p	ORa (IC 95%)	p
**Age class**						
≤ 35 years old	125 (66.5%)	2.3 (±2,5)	1		1	
> 35 years old	79(62.2%)	2.7 (±2.9)	0.8 (0.5-1.3)	<0.435	1.08 (0.6-1.84)	<0.783
**Studies**	157(73%)					
None/ primary	47 (47%)	3 (±2.8)	1		1	
Secondary or		1.3 (±1.9)	0.33 (0.20-0,54)	<0.001	0.59 (0.33-0.93)	<0.048
University						
**Paid occupation**					-	
No	101 (66.9%)	2.7 (2.8)	1			
Yes	10 (62.8%)	2.2 (2.5)	0.83 (0.52-1.33)	<0.449		
**Profession**					-	
Worker	61 (70.1%)	2.7 (2.7)	1			
Intermediary/ prof	26 (63.4%)	2.2 (2.5)	0.7 (0.3-1.6)	<0.027		
Manager	16 (44.4%)	1 (1.4)	0.3 (0.2-0.8)			
**Social support**					-	
Weak support	117 (65.7%)	2.2 (2.5)	1			
Strong support	87 (63.5%)	2.7 (2.8)	0.91 (0.57-1.44)	<0.682		
**Contacts frequency**	54 (79.4%)					
Less than once/month	83 (71.6%)	3.3 (2.9)	1		1	
At least once/month	67 (51.2%)	2.5 (2.6)	0.6 (0.3-1.3)	**<**0.001	0.72 (0.34-1.54)	<0.401
At least once/week		1.9 (2.5)	0.3 (0.2-0.5)		0.32 (0.15-0.67)	<0.002
**Level of wealth**						
Poor	127 (73.8%)	1.23 (1.85)	1		1	
Average	57 (60%)	2.02 (2.50)	0.5 (0.3-0.9)	<0.001	0.57 (0.29-1.12)	<0.104
Wealth	20 (41.7%)	3.05 (2.81)	0.3 (0.1-0.5)		0.23 (0.08-0.64)	<0.005
**Structure of household**						
Extended	42 (69.9%)	2.69 (2.68)	1		1	
Nuclear	53(57.6%)	2.12 (2.61)	0.6 (0.4-0.9)	<0.021	0.76 (0.43-1.31)	<0.323
Reconstituted	9(45%)	1.75 (1.70)	0.4 (0.2-0.9)		0.40 (0.15-1.08)	<0.072
**Density/occupation**						
Dense	143(69.1%)	2.8 (2.8)	1		1	
Less dense	61(56.5%)	1.8 (2.1)	0.6 (0.4-0.9)	<0.027	0.61 (0.29-1.27)	<0.189

SD: standard deviation; ORna: non-adjusted ratio-odds; Ora: adjusted odds ratio; IC95%: interval of confidence up to 95%; Intermediary/ prof: intermediary/ profession

## Discussion

This study mainly, suggests, the existence of dental caries disparities among the Pikine´s children associated with the specific characteristics of mothers and to social conditions of life in their households. Precisely, it noticed a significant upward trend of the children dental caries prevalence with the mother´s lower studies level and social network dynamism, as well as with the low wealth level or the size family population. The study presents strengths and limitations. Among the strengths, clinical data was obtained by children´s examination. The sample size is another strength of the study which is sufficiently powerful to discriminate the population according to the studied parameters. The study also shows some limitations that do not affect the result's credibility. The main limits of the study are its cross-sectional design that is not adapted to the comparison. Another limitation is the estimation of the level of wealth which is rather subjective. Our results are in agreement with previous research [27-29]. The mother´s socio-economic characteristics, approximate by studies level, profession and social network have an indirect effect on the risk of decay in children. Indeed, an abundant literature has previously proved the inverse relation between the prevalence of children´s dental caries and the mother´s studies level [30-32]. There are mediation ways through which the mother´s studies level has impact on children´s caries disparities. Among these, the level of knowledge and attitude of mother toward dental health are determinant [33]. A high level of studies contributes to the improvement of knowledge in oral health [34], oral health literacy and a positive attitude of mothers [35]; such factors predict a low prevalence of children´s dental caries [36]. In the context of Pikine, this conclusion can be explained by the low rate of schooling in the women which is more pronounced in disadvantaged lower classes.

A trend test showed a significant increasing of the prevalence of children´s caries, conversely, in the professional status of mothers. The professional status is a proxy for incomes level. The workers have fewer incomes than managers with potential implications on oral health inequalities among children [18]. In contrast to these results, a study in Eastern Saudi-Arabia reported a higher risk of dental caries among children whose mothers had a higher professional status. They associate this finding with the mothers´ lack of time to take care of the children´s oral health [37]. This explanation does not apply in the Senegalese context, in particular in the suburbs, where the children are taken care of within the framework of the family by stepmothers, aunts among others. Children do not suffer from lack of care because of the work of their mothers. The study showed an association between the mothers´ social network and social inequalities in children´s dental caries. The more active the mothers´ social network is, the more the prevalence of children tooth decay decreases. The social network contribution in oral health is to provide informational and emotional resources [38]. A recent study by Burgette *et al*. found a significant association between social supports (approached by social interactions) perceived by mothers and children´s dental caries [39]. Lida and Rozier reported that mothers who had higher social capital had children with better oral health [40]. Both social support and social, as understood in previous studies, integrate the notion of social network in its dimension of intensity of social interactions. Active social network constitutes for mothers a place of exchange and sharing information and knowledge. The members can be informed of the existence of a preventive oral health program for children. They can also further expand their oral health knowledge [39,41].

However, in the popular neighborhoods of Pikine, many women gather into networks of solidarity, “informal” entrepreneurship, and very active cultural or religious organizations. The mothers´ individual characteristics are fitted in the family social context. In this study, the family social context is characterized by the material resources (or wealth) and the household structure. The results of a logistic regression model suggest a gradient of prevalence of children´s dental caries according to wealth level and family structure. The trend in the risk of children´s dental caries is decreasing from poor households to wealth households and from extended-type households to nuclear ones. Previous studies had shown an association between higher prevalence of children´s dental caries and material conditions of precarious family as well as high household overcrowding [30,42]. Costa *et al*. argue that incomes which doesn´t cover the family needs into hygienically oral and dental products were predictive to severe caries diseases [43]. On the other hand, children whose families have at their disposal enough incomes were less at risk to develop dental diseases [44]. This observation can be explained by the materialist theory of health social inequalities. It suggests that people from a lower socioeconomically status, especially children, are more sensitive to many factors of risks to illnesses, such as inadequate food and lack of resources [45]. The National agency of statistics and demography of Senegal reported that the households in the department of Pikine were the poorest ones of Dakar and the feeling of social welfare of women was among the weakest [26]. Therefore, this precariousness can lead parents to a hedging on available weak incomes which is generally made to the detriment of oral health.

The results also suggest the existence of a gradient in the prevalence of caries according to the family structure. The prevalence is low in nuclear families compared to extended families. Extended families have generally more members than nuclear ones. Previous studies had underlined that financial and social pressures associated with family size (large family) often have a negative impact on children´s oral health [46]. Indeed, the difficult social situation of households in Pikine can make it impossible for them to satisfy the needs of all members with its emotional consequences on mothers. This state of fact can favor a failure to control risk behaviors or preventive attitudes towards children's caries. We therefore consider the limit on wealth evaluation to be minor, because to assess household poverty, the government uses, as we have done in this study, the asset list system. It should be noted that the estimation of the level of wealth is difficult in our skies where there is a modesty among the populations to speak about money. Pikine from which these results are taken is a suburb of Dakar characterized by overcrowding, a deficit of basic social services and difficult social conditions. This social reality is similar to many rural areas and suburbs of a lot of cities in sub-Saharan Africa. Thus, these results are generalizable in these places. However, in residential areas, reality does not allow such results to be taken into account in the development of public health actions. This study offers evidence to aid policy decisions in the direction of reducing inequality and social injustice.

## Conclusion

The results of this study highlighted the extent of social inequalities in oral health in children, at an age when the actions of health promotion have more likely to bring positive results. It puts into perspective the possibilities of intervention in a proportionate universalism approach. It consists of offering some basic operations to all children/families and according to the needs relating to risks and the social precariousness, proposing additional interventions by weighting the intensity. It will be about encouraging the set-up of local or national social policies such the strengthening basic social services, extending health risk coverage among others. The turning to profit of a dynamic network of community workers which has been successful in maternal health is important to be explored for the benefit of target groups.

### What is known about this topic

- Theoretical framework of the individual and family determinants of children's dental caries;
- The association between social conditions of households and oral health of children.

### What this study adds

- Although the social inequalities of dental caries are studied in children, they are very little related to maternal characteristics in sub-Saharan Africa; so, the study brings, among other new knowledge the impact of mothers’ individual characteristics on social inequalities in dental caries in a precarious context;
- The relationship of the ways of socialization, especially social network or social support, with the occurrence or maintenance of dental caries social inequalities of children in a country with limited resources;
- Maternal social determinants of social inequalities in dental caries in Senegalese children.
